# Safety and efficacy of intravenous thrombolysis before mechanical thrombectomy in patients with atrial fibrillation

**DOI:** 10.1186/s13643-024-02532-1

**Published:** 2024-04-30

**Authors:** Qiangji Bao, Xiaodong Huang, Xinting Wu, Hao Huang, Xiaoqiang Zhang, Mingfei Yang

**Affiliations:** 1 Department of Neurosurgery, Guang’an People’s Hospital, Guang’an, Sichuan, China; 2 Department of Neurosurgery, Taihe Hospital Affiliated Hospital of Hubei University of Medicine, Shiyan, Hubei China; 3 Department of Anesthesia, Guang’an People’s Hospital, Guang’an, Sichuan, 638000 China; 4 Department of Neurosurgery, Qinghai Provincial People’s Hospital, Xining, Qinghai 810007 China

**Keywords:** Ischemic stroke, Large vessel occlusion, Atrial fibrillation, Intravenous thrombolysis, Mechanical thrombectomy

## Abstract

**Background:**

Intravenous thrombolysis (IVT) before endovascular thrombectomy (EVT) is the standard treatment for patients with acute ischemic stroke caused by large vessel occlusion (AIS-LVO). However, the efficacy and safety of IVT before EVT in AIS-LVO patients with atrial fibrillation (AF) remains controversial. Thus, this study aims to assess the benefit of IVT plus EVT and direct EVT alone in AIS-LVO patients with AF.

**Method:**

Relevant studies that evaluated the outcomes of IVT plus EVT versus direct EVT alone in AIS-LVO patients with AF were systematically searched in PubMed, Embase, and Cochrane Library from inception to August 10, 2023. The outcomes included successful reperfusion (score of 2b to 3 for thrombolysis in cerebral infarction), symptomatic intracerebral hemorrhage (sICH), good clinical outcome (modified Rankin scale score ≤ 2) at 3 months, and 3-month mortality.

**Result:**

Eight eligible observational studies involving 6998 (3827 in the IVT plus EVT group and 3171 in the direct EVT group) patients with AIS-LVO complicated by AF were included. Compared with direct EVT, IVT plus EVT resulted in better 3-month clinical outcomes (odds ratio [OR] 1.27, 95% confidence interval [CI] 1.05–1.54) and lower 3-month mortality (OR 0.78, 95% CI 0.68–0.88). However, the incidence of sICH (OR 1.26, 95% CI 0.91–1.75) and the rate of successful reperfusion (OR 0.98, 95% CI 0.83–1.17) were not significantly different between treatment modalities.

**Conclusion:**

IVT plus EVT leads to better functional outcomes and lower mortality in AIS-LVO patients with AF. Withholding IVT plus EVT from patients with AF alone may not be justified.

**Supplementary Information:**

The online version contains supplementary material available at 10.1186/s13643-024-02532-1.

## Introduction

Acute ischemic stroke (AIS) is the primary cause of disability and mortality worldwide [1]. Recanalization is essential for salvaging the ischemic penumbra and improving the overall prognosis of AIS. Intravenous thrombolysis (IVT) is the first reperfusion therapy that is effective for AIS [2]. Several pivotal randomized-controlled trials in 2015 [3–8] demonstrate that endovascular thrombectomy (EVT) is more effective than IVT in improving the prognosis of patients suffering from AIS due to a large vessel occlusion (LVO). Guidelines have recommended IVT combined with EVT for patients who meet the criteria for both treatment modalities [9–11].

Several recent studies have shown conflicting results when comparing the outcomes of IVT plus EVT versus EVT alone. For example, two randomized controlled trials (RCTs) [12, 13] have demonstrated that direct EVT is not inferior to IVT plus EVT in eligible patients, whereas other RCTs [14–17] have failed to establish non-inferiority or have suggested inferiority. This might be attributable to heterogeneity in the stroke population and the distinct reactions to IVT plus EVT. Hence, it is crucial to consider individual patient characteristics and variables when determining the optimal reperfusion strategy.

Atrial fibrillation (AF) is a prevalent cardiac arrhythmia and a significant risk factor for cardioembolic stroke, contributing to one-third of AIS cases worldwide [18]. It is associated with a five-fold increase in the incidence of AIS, leading to an inferior functional outcome and increased mortality in ischemic stroke patients [19]. The efficacy of IVT before EVT in patients with AF remains controversial [20]. Additionally, IVT before EVT may increase the risk of bleeding, particularly in AF patients receiving anticoagulant therapy [21]. Therefore, it is crucial to consider the potential benefits and risks of IVT before EVT in this patient population.

Given the clinical specificity and absence of randomized data on AIS-LVO patients with AF, we conducted a systematic review and meta-analysis to assess the safety and efficacy of IVT plus EVT and direct EVT in this patient population.

## Methods

### Data availability statement

All data generated or analyzed during this study are included in this article (and/or) in its supplemental materials.

### Standard protocol approval, registration, and patient consent

This study was conducted following the Preferred Reporting Items for Systematic Reviews and Meta-Analyses (PRISMA) statement [22], and the study protocol (INPLASY202390015) has been registered with the International Protocol Registration Platform for Systematic Reviews and Meta-Analyses (INPLASY, https://inplasy.com/). This study does not require ethics committee approval or written informed consent from patients.

### Data sources and study selection

RCTs or observational cohort studies were systematically searched in PubMed, EMBASE, and Cochrane Library using the keywords “stroke,” “atrial fibrillation,” “intravenous thrombolysis,” and “mechanical thrombectomy.” Studies were selected based on the following PICO (patients, interventions, comparators, and outcomes) criteria: (1) patients, AIS- LVO combined with AF; (2) intervention, IVT plus EVT; (3) comparator, direct EVT; and (4) outcomes, 3-month good clinical outcome (modified Rankin score of 0–2 [23]), symptomatic intracerebral hemorrhage (sICH), successful reperfusion (thrombolysis in cerebral infarction (TICI) scores of 2b to 3 [23]), and 3-month mortality.

The literature search was independently performed by 2 investigators (BQJ and HXD). The full search strategy is shown in Supplementary eTable 1.

### Data extraction

Two investigators (BQJ and HXD) independently extracted data using a standardized form. Data extracted include data source, type of study, study duration, sample size, age, sex, baseline National Institutes of Health Stroke Scale (NIHSS), Alberta Stroke Program Early CT Score (ASPECTS), high blood pressure, diabetes, dyslipidemia, previous stroke, previous cardiovascular disease, smoking history, time from onset to admission, time from onset to puncture, time from puncture to reperfusion, first author’s name, year of publication, and primary endpoint(s).

### Quality and risk of bias assessments

The risk of bias in each study was critically assessed by two independent investigators (BQJ and HXD) using the Newcastle–Ottawa Scale (NOS) [24]. Any discrepancies were resolved by discussion with the corresponding author (ZXQ). All studies were scored for selection, comparability, and outcomes. A study with an NOS score of 7 or higher is considered high quality.

### Statistical analysis

In pairwise meta-analyses, the corresponding odds ratios (ORs) and 95% confidence intervals (95% CIs) for the outcome events were calculated for the direct EVT and IVT plus EVT groups. Pooled estimates were determined using a random effects model (DerSimonian and Laird) [25]. Statistical heterogeneity across trials was assessed by the Cochran Q test with a significance level of *p* < 0.1 and quantified by the *I*^*2*^ statistic [26], with an *I*^2^ value greater than 50% indicating substantial heterogeneity. Publication bias was evaluated using funnel plots. All statistical analyses were performed using Review Manager (RevMan v.5.3).

## Results

### Literature search and screening

**Fig. 1:**
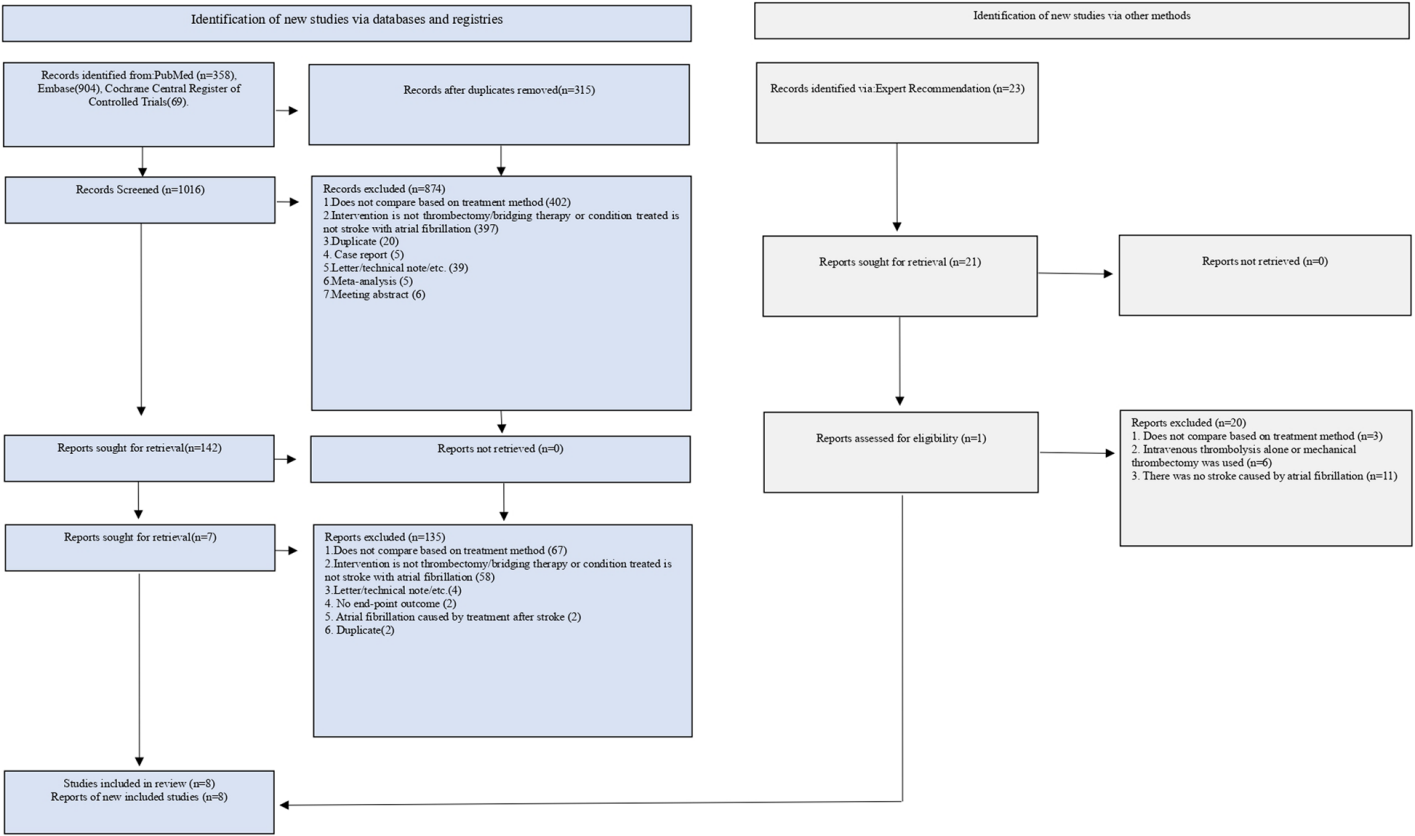
Flow chart of literature search and study selection

Of the initially identified 1331 articles, 315 were removed due to duplication, and 1010 were excluded due to ineligibility. Six records were retained for full-text screening, and two additional studies were identified by expert advice. Ultimately, 8 studies [27–34], involving 6798 patients, met our inclusion criteria and reported relevant outcomes (Fig. 1).

### Study characteristics and risk of bias

Among the eight included studies, six [27–29, 31–33] were retrospective observational studies, and two [30, 34] were prospective observational studies. The number of patients per study ranged from 94 to 2311. Four studies [28, 29, 31, 34] were determined to be of good quality, and the other four studies [27, 30, 32, 33] were of acceptable quality (Supplementary eTable 2). Characteristics of eligible studies are summarized in Table 1.

**Table 1:** Descriptive characteristics of the included studies

Study	Loo et al, 2023 [27]	Lin et al, 2023 [28]	Cao et al, 2022 [29]	Chen et al, 2022 [30]	Mujanovic et al, 2022 [31]	Akbik et al, 2022 [32]	Yaghi et al, 2021 [33]	Chalos et al, 2019 [34]
Data source	Multicenter study	INSPIRE	DIRECT-MT study	Single-center study	BEYOND-SWIFT	STAR	ICA	MR CLEAN
Study type	RO of prospective database	RO of prospective database	RO	PO	RO of prospective database	RO of prospective database	RO of prospective database	PO
Start to end of recruitment period	2015.1-2021.12	2016.1-2019.12	2018.5-2020.5	2015.1-2021.12	2015-2018	2015.6-2020.12	2015-2018	2014.3-2016.6
Sample size- direct mechanical thrombectomy vs bridging therapy	132 vs 182	223 vs 212	146 vs 144	52 vs 42	715 vs 632	1275 vs 1036	290 vs 232	324 vs 1161
Age (years) (dMT vs BT, mean±SD or median,IQR)	73.6 (10.9) vs 73.2 (10.3)	76.0 (69.0–83.5) vs 75.5 (69.0–83.0)	73.0 (65.0-76.0) vs 71.0 (66.0-75.0)	68.0 (10.0) vs 69.0 (9.0)	78.0 (70.0-84.0) vs 77.0 (68.0- 83.0)	76.0 (11.0) vs 76.0 (11.0)	77.0 (11.5) vs 77.6 (12.0)	72.0 (63.0–80.0) vs 70.0 (59.0–79.0)
Gender (dMT vs BT, Female) (n/N (%))	69/132 (52.3) vs 108/182 (59.8)	128/333 (54.9) vs 103/211 (48.8)	78/146 (53.4)vs 82/144 (56.9)	23/52 (44.2) vs 17/42 (40.4)	423/715 (59.2) vs 338/632 (53.5)	688/1275 (54.0) vs 559/1036 (54.0)	126/290 (43.4) vs 104/232 (44.8)	171/324 (53.0) vs 621/1161 (54.0)
Baseline NIHSS(dMT vs BT, mean±SD or median,IQR)	8.5 (18.4) vs 8.1 (18.3)	17.0 (12.0–21.0) vs 18.0 (13.0–21.0)	18.0 (14.0-23.0) vs 19.0 (14.0-23.0)	20.0 (17.0-25.5) vs 18.5 (16.0-22.0)	17.0 (11.0-20.0) vs 16.0 (11.0-20.0)	16.0 (7.0) vs 16.0 (6.0)	17.0 (11.0–22.0) vs 18.0 (13.0–23.0)	17.0 (13.0–20.0) vs 16.0 (11.0–20.0)
ASPECTS(dMT vs BT, median,IQR)	9 (8-10) vs 9 (8-10)	NR	9 (7-10) vs 9 (7-10)	NR	9 (8-10) vs 9 (8-10)	ASPECT score >6: 86% vs 87%	9 (7-10) vs 9 (7-10)	9 (7–10) vs 9 (7–10)
HTN (dMT vs BT, n/N (%))	102/132 (77.3) vs 147/182 (80.8)	174/233 (74.7) vs 152/212 (71.7)	92/146 (63.0) vs 97/144 (67.4)	30/52 (57.7) vs 21/42 (50.0)	548/715 (77.0) vs 441/632 (70.2)	1070/1275 (84.0) vs 840/1036 (81.0)	236/288 (81.9) vs 186/232 (80.2)	180/321 (56.0) vs 562/1145 (49.0)
DM (dMT vs BT, n/N (%))	38/132 (24.2) vs 44/182 (28.8)	56/233 (24.0) vs 38/210 (18.1)	32/146 (21.9) vs 29/144 (20.1)	11/52 (21.2) vs 11/42 (26.2)	154/715 (21.7) vs 131/632 (20.9)	389/1273 (31.0) vs 293/1029 (29.0)	80/287 (27.9) vs 55/231 (23.8)	56/321 (17.0) vs 197/1155 (17.0)
Dyslipidemia (dMT vs BT, n/N (%))	50/132 (37.9) vs 81/182 (44.5)	70/217 (32.3) vs 55/196 (28.1)	NR	NR	330/715 (46.6) vs 288/632 (46.1)	633/1273 (50.0) vs 442/1034 (43.0)	156/288 (54.2) vs 106/232 (45.7)	NR
Prior stroke (dMT vs BT, n/N (%))	33/132 (25.0) vs 14/182 (7.7)	35/207 (16.9) vs 43/201 (21.4)	24/146 (16.4) vs 24/144 (16.7)	7/52 (13.5) vs 5/42 (11.9)	118/715 (19.3) vs 56/632 (11.2)	246/1083 (23.0) vs 123/818 (15.0)	83/288 (28.8) vs 54/232 (23.3)	83/322 (26.0) vs 164/1154 (14.0)
Prior CVD (dMT vs BT, n/N (%))	28/132 (21.2) vs 38/182 (20.9)	NR	NR	10/52 (19.2) vs 6/42 (14.3)	NR	NR	95/289 (32.9) vs 60/231 (26.0)	36/318 (11) vs 99/1139 (8.7)
Smoking (dMT vs BT, n/N (%))	16/132 (12.1) vs 16/182 (8.8)	35/207 (16.9) vs 43/201 (21.4)	NR	6/52 (11.5) vs 6/42 (14.3)	112/715 (16.2) vs 111/632 (18.5)	NR	31/250 (12.4) vs 29/207 (14.0)	NR
Alteplase dose	0.9mg/kg	NR	0.9mg/kg	0.9mg/kg	NR	0.9mg/kg	NR	0.9mg/kg
Time from onset to admission(dMT vs BT)	0-6 vs 0-4.5 h	NR	167 (125-206) vs 177 (126-215) min	0-4.5 vs 0-4.5 h	NR	NR	NR	NR
Time from onset to puncture(dMT vs BT, median,IQR or mean±SD)	210 (160–274) vs 195 (158–250) min	4.70 (3.42–6.76) vs 4.70 (3.42–6.76) h	218 (170–254) vs 197 (169–268) min	210±78 vs 218±76 min	NR	51 (41) vs 48 (39) min	NR	47 (31–69) vs 47 (30–71) min
Time from puncture to reperfusion (dMT vs BT, median, IQR or mean±SD)	36 (20–60) vs 38 (20–62) min	NR	31 (20-45) vs 36 (20-50.5) min	(70±37) vs (78±38) min	NR	4.3(3.0) vs 7.7 (7.0) h	NR	215 (158–294) vs 206 (160–260) min
End-point	①, ②, ③, ④	①, ②, ③, ④	①, ②, ③, ④	①, ②, ③, ④	①, ③, ④	①, ②, ③, ④	②, ④	①, ②, ③, ④

*dMT* direct mechanical thrombectomy, *BT* bridging therapy, *HT*N hypertension, *DM* diabetes mellitus, *CVD* cardiovascular disease, *NR* no report, *PO* prospective observational, *RO* retrospective observational;

*INSPIRE* International Stroke Perfusion Imaging Registry, *BEYOND-SWIFT* The Bernese-European Registry for Ischemic Stroke Patients Treated Outside Current Guidelines With Neurothrombectomy Devices Using the Solitaire FR with the Intention for Thrombectomy, *STAR* Stroke Thrombectomy and Aneurysm Registry, *ICA* Initiation of Anticoagulation in Cardioembolic Stroke

①: 3-month good clinical outcome (defined as an modified Rankin Score of 0-2); ②: Successful reperfusion (defined as thrombolysis in cerebral infarction scores of 2b to 3); ③: symptomatic intracerebral hemorrhage ; ④: 3-month mortality

### 3-Month good clinical outcome

**Fig. 2:**
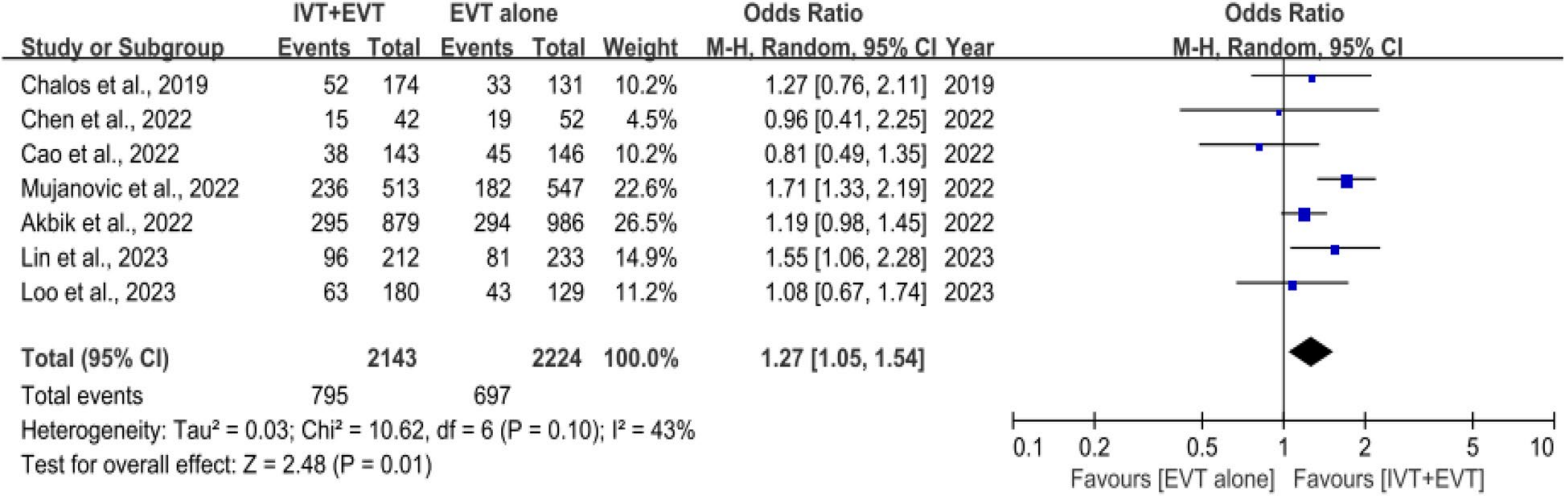
Forest plot of rates of good clinical outcome (defined as an mRS Score of 0-2)

Seven studies [27–32, 34] compared the good clinical outcome of 6267 AIS-LVO patients with AF. Our data showed that IVT plus EVT was associated with good clinical outcomes at 3 months compared with EVT alone (OR, 1.27 [95% CI, 1.05–1.54]; *P* = 0.01) (Fig. 2). No significant heterogeneity was observed among studies (*I*^*2*^ = 43%; *P* = 0.10).

### Successful reperfusion rate

**Fig. 3:**
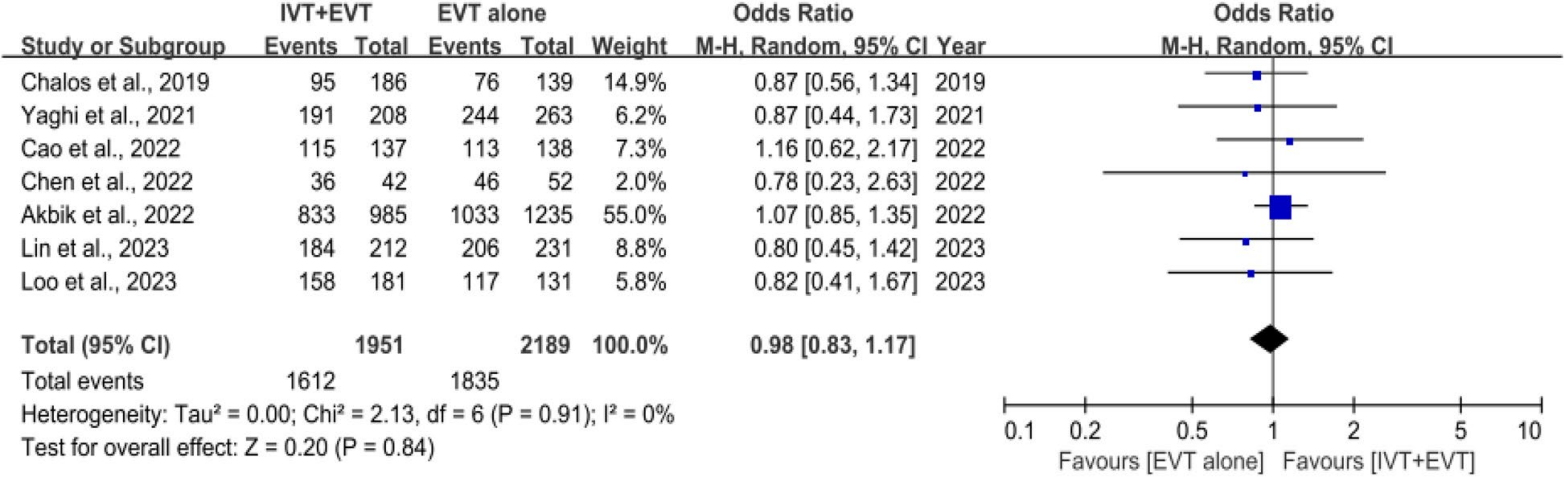
Forest plot of rates of successful reperfusion (defined as thrombolysis in cerebral infarction scores of 2b to 3)

Successful reperfusion rates were reported in 7 studies [27–30, 32, 34] involving 5,451 AIS-LVO patients with AF. The successful reperfusion rate was not significantly different between IVT plus EVT and direct EVT (OR, 0.98 [95% Cl, 0.83–1.17]; *P* = 0.84), and no heterogeneity was observed across studies (*I*^2^ = 0%,* P* = 0.91) (Fig. 3).

### sICH

**Fig. 4:**
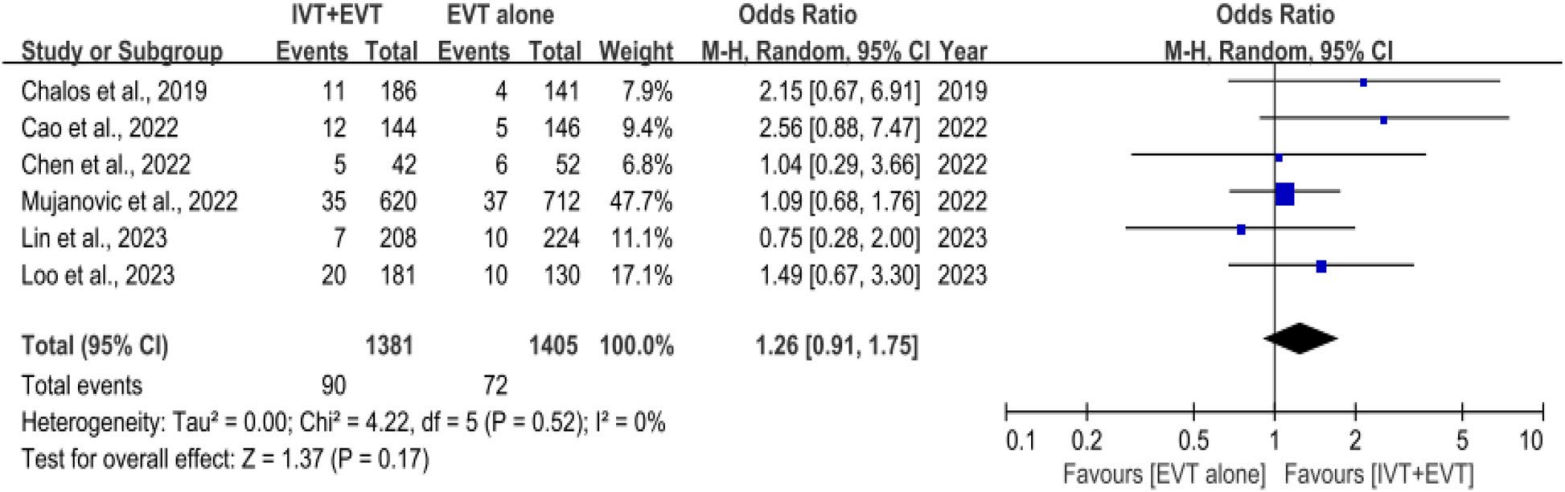
Forest plot of rates of symptomatic intracerebral hemorrhage

Six studies [27–31, 34] involving 3965 patients compared the incidence of sICH with and without IVT. The incidence of sICH showed no significant difference between the two groups (OR, 1.26 [95% CI, 0.91–1.75]; *P* = 0.17), and there was no between-study heterogeneity (*I*^2^ = 0%, *P* = 0.52) (Fig. 4).

### 3-Month mortality

**Fig. 5:**
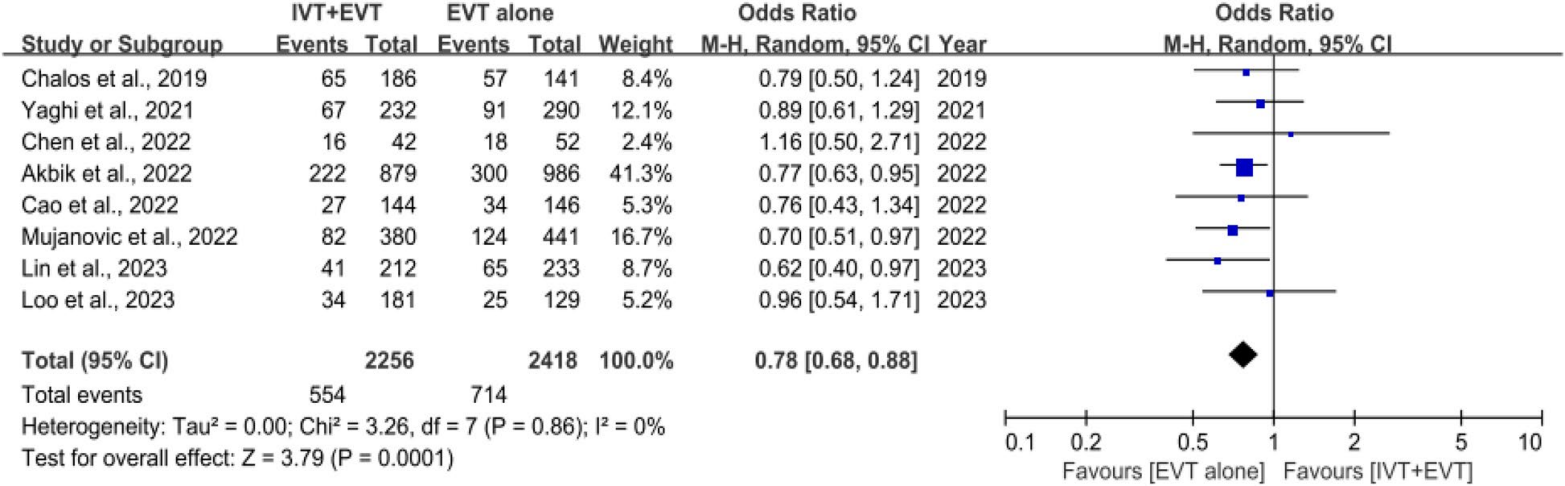
Forest plot of rates of mortality

Eight studies involving 6798 patients [27–34] compared 3-month mortality with and without IVT. The IVT plus EVT group had a significantly lower mortality rate than the direct EVT group (OR, 0.78 [95% Cl, 0.68–0.88]; *P* = 0.0001), and no heterogeneity was observed among studies (*I*^2^ = 0%; *P* = 0.86) (Fig. 5).

## Discussion

Our meta-analysis revealed that IVT plus EVT results in better clinical outcomes and lower mortality rates compared with direct EVT. The incidence of sICH and the rate of successful reperfusion were not significantly different between the two groups. In the absence of randomized data, this is the first study to systematically assess the efficacy and safety of IVT before EVT in AIS-LVO patients with AF.

For patients with AIS-LVO who are eligible for IVT and EVT, relevant guidelines recommend the initiation of combination therapy within 4.5 h of stroke onset [9–11]. Thrombolysis can enhance the efficiency of embolus dissolution, facilitate embolus removal, and reduce both the recanalization time and the rate of EVT. Furthermore, TICI 2C or 3 reperfusion has been shown to exhibit superior functional prognosis compared with TICI 2B reperfusion [35]. AF is associated with a decreased frequency of venous thrombosis recanalization [36, 37]. When managing AF, the impaired efficacy of venous thrombosis is believed to be caused by decreased collateralization, limiting the penetration of thrombolytic medications into the thrombus [38]. This, in turn, results in unfavorable clinical outcomes in AF patients who undergo bridging therapy. Our data demonstrated that IVT plus EVT led to a comparable successful reperfusion rate but greater good clinical outcomes and lower mortality rates than direct EVT. This is likely attributed to the increased density of blood clots due to the presence of red blood cells and interstitial fibrin. Both types of clots are dense, but they penetrate recombinant tissue plasminogen activators more easily than leukocyte-rich clots that contain densely packed platelets and cell debris. Since red blood cell clots typically occur in cardiac embolism and are more likely to lead to late spontaneous recanalisation [39], this may explain the lack of a significant association with successful reperfusion.

While we did not observe any advantage of IVT plus EVT in successful reperfusion rate compared with direct EVT, we found that pre-thrombolysis enhanced functional outcomes and reduced mortality. Cao et al. revealed that the rate of recanalization before intravascular therapy was notably higher in the bridging thrombolytic group than in the untreated group [29]. Consistent with our findings, Zhou et al. have reported that individuals who undergo early reperfusion show improved clinical outcomes, suggesting that a certain level of reperfusion should be achieved before EVT to optimize patient prognoses [40]. Furthermore, thrombolytic drugs are the most efficacious pharmacological therapy for AIS, greatly improving survival rates and reducing disabilities among cerebral infarction patients [41, 42].

Intracranial hemorrhage is the most serious complication of thrombolysis in stroke and an important obstacle in the wide application of thrombolytic therapy [43]. AF is an independent risk factor for hemorrhagic transformation following thrombolysis [44]. We found that prior IVT did not elevate the incidence of symptomatic bleeding in AF patients who subsequently underwent EVT. This evidence confirms the safety of IVT plus EVT.

The Stroke Guidelines in the USA, Europe, and China all recommend thrombolysis within 4.5 h of onset without impacting EVT [9–11]. Therefore, it is not justifiable to categorize AF patients based solely on the study by Akbik et al. [32], as this may limit the number of eligible patients for bridging thrombolysis therapy, subsequently hindering their chances of benefiting from the treatment. Our findings contribute to the redefinition of the clinical practices for bridging thrombolytic therapy in AIS-LVO patients with AF and lay the groundwork for future efforts. Nonetheless, the inconsistency in our results has undermined confidence in bridging thrombolytic therapy, and further RCTs are warranted to verify its efficacy.

## Limitations

First, this is a post hoc analysis that solely relied on the retrospective analyses of prospective observational and retrospective observational. As a result, the results are susceptible to confounding factors. Second, due to limited available data, we were unable to further examine the differences between patients with and without AF. Third, treatment allocation was determined by treating neurologists, and functional outcomes were assessed by physicians blinded to treatment details, potentially introducing selection and confirmation biases, respectively. Last, we did not conduct an in-depth analysis of the effects of anticoagulants.

## Conclusion

IVT plus EVT is a promising treatment for stroke patients with AF without raising the risk of sICH. Consequently, it is unjustified to dismiss thrombolytic treatment solely based on the presence of AF.

### Supplementary Information

**Supplementary Material 1.**

## Data Availability

All data generated or analyzed during this study are included in this article (and/or) its supplementary material files. Further inquiries can be directed to the corresponding author.

## References

[1] GBD 2016 Stroke Collaborators (2019). Global, regional, and national burden of stroke, 1990–2016: a systematic analysis for the Global Burden of Disease Study 2016. Lancet Neurol.

[2] National Institute of Neurological Disorders and Stroke rt-PA Stroke Study Group (1995). Tissue plasminogen activator for acute ischemic stroke. N Engl J Med.

[3] Bracard S, Ducrocq X, Mas JL (2016). Mechanical thrombectomy after intravenous alteplase versus alteplase alone after stroke (THRACE): a randomised controlled trial. Lancet Neurol.

[4] Campbell BC, Mitchell PJ, Kleinig TJ (2015). Endovascular therapy for ischemic stroke with perfusion-imaging selection. N Engl J Med.

[5] Saver JL, Goyal M, Bonafe A (2015). Stent-retriever thrombectomy after intravenous t-PA vs t-PA alone in stroke. N Engl J Med.

[6] Jovin TG, Chamorro A, Cobo E (2015). Thrombectomy within 8 hours after symptom onset in ischemic stroke. N Engl J Med.

[7] Tao C, Nogueira RG, Zhu Y (2022). Trial of endovascular treatment of acute basilar-artery occlusion. N Engl J Med.

[8] Nogueira RG, Jadhav AP, Haussen DC (2018). Thrombectomy 6 to 24 hours after stroke with a mismatch between deficit and infarct. N Engl J Med.

[9] Liu L, Chen W, Zhou H (2020). Chinese Stroke Association guidelines for clinical management of cerebrovascular disorders: executive summary and 2019 update of clinical management of ischaemic cerebrovascular diseases. Stroke Vasc Neurol.

[10] Powers WJ, Rabinstein AA, Ackerson T (2019). Guidelines for the Early Management of Patients With Acute Ischemic Stroke: 2019 Update to the 2018 Guidelines for the Early Management of Acute Ischemic Stroke: A Guideline for Healthcare Professionals From the American Heart Association/American Stroke Association. Stroke.

[11] Turc G, Bhogal P, Fischer U (2019). European Stroke Organisation (ESO)- European Society for Minimally Invasive Neurological Therapy (ESMINT) guidelines on mechanical thrombectomy in acute ischemic stroke. J Neurointerv Surg.

[12] Yang P, Zhang Y, Zhang L (2020). Endovascular thrombectomy with or without intravenous alteplase in acute stroke. N Engl J Med.

[13] Zi W, Qiu Z, Li F (2021). Effect of endovascular treatment alone vs intravenous alteplase plus endovascular treatment on functional independence in patients with acute ischemic stroke: the DEVT randomized clinical trial. JAMA.

[14] Suzuki K, Matsumaru Y, Takeuchi M (2021). Effect of mechanical thrombectomy without vs with intravenous thrombolysis on functional outcome among patients with acute ischemic stroke: the SKIP randomized clinical trial. JAMA.

[15] Mitchell PJ, Yan B, Churilov L (2022). Endovascular thrombectomy versus standard bridging thrombolytic with endovascular thrombectomy within 4·5 H of stroke onset: an open-label, blinded-endpoint, randomised non-inferiority trial. Lancet.

[16] LeCouffe NE, Kappelhof M, Treurniet KM (2021). A randomized trial of intravenous alteplase before endovascular treatment for stroke. N Engl J Med.

[17] Fischer U, Kaesmacher J, Strbian D (2022). Thrombectomy alone versus intravenous alteplase plus thrombectomy in patients with stroke: an open-label, blinded-outcome, randomised non-inferiority trial. Lancet.

[18] Wolf PA, Abbott RD, Kannel WB (1991). Atrial fibrillation as an independent risk factor for stroke: the Framingham study. Stroke.

[19] Steger C, Pratter A, Martinek-Bregel M (2004). Stroke patients with atrial fibrillation have a worse prognosis than patients without: data from the Austrian Stroke Registry. Eur Heart J.

[20] Bozzani A, Arici V, RagniIntravenous F (2023). thrombolysis before mechanical thrombectomy in patients with atrial fibrillation. J Neurointerv Surg.

[21] Stanton RJ, Eckman MH, Woo D (2020). Ischemic stroke and bleeding: clinical benefit of anticoagulation in atrial fibrillation after intracerebral hemorrhage. Stroke.

[22] Page MJ, McKenzie JE, Bossuyt PM (2021). The PRISMA 2020 statement: an updated guideline for reporting systematic reviews. BMJ..

[23] Turc G, Tsivgoulis G, Audebert HJ (2022). European Stroke Organisation (ESO)-European Society for Minimally Invasive Neurological Therapy (ESMINT) expedited recommendation on indication for intravenous thrombolysis before mechanical thrombectomy in patients with acute ischemic stroke and anterior circulation large vessel occlusion. J Neurointerv Surg.

[24] Stang A (2010). Critical evaluation of the Newcastle-Ottawa scale for the assessment of the quality of nonrandomized studies in meta-analyses. Eur J Epidemiol.

[25] DerSimonian R, Laird N (2015). Meta-analysis in clinical trials revisited. Contemp Clin Trials.

[26] Cumpston M, Li TJ, Page MJ, Chandler J (2019). Updated guidance for trusted systematic reviews: a new edition of the Cochrane Handbook for Systematic Reviews of Interventions. Cochrane Database Syst Rev..

[27] Loo JH, Leow AS, Jing MX (2023). Impact of atrial fibrillation on the treatment effect of bridging thrombolysis in ischemic stroke patients undergoing endovascular thrombectomy: a multicenter international cohort study. J Neurointerv Surg..

[28] Lin LT, Blair C, Fu J (2023). Prior anticoagulation and bridging thrombolysis improve outcomes in patients with atrial fibrillation undergoing endovascular thrombectomy for anterior circulation stroke. J Neurointerv Surg..

[29] Cao J, Xing PF, Zhu XC (2022). Mild and moderate cardioembolic stroke patients may benefit more from direct mechanical thrombectomy than bridging therapy: a subgroup analysis of a randomized clinical trial (DIRECT-MT). Front Neurol.

[30] Chen YX, Guo Y, Lin YN (2022). A comparative study between direct mechanical thrombectomy and bridging therapy in patients with atrial fibrillation associated anterior circulation large vessel occlusion stroke. Zhonghua Yi Xue Za Zhi.

[31] Mujanovic A, Kurmann CC, Dobrocky T (2022). Bridging intravenous thrombolysis in patients with atrial fibrillation. Front Neurol.

[32] Akbik F, Alawieh A, Dimisko L (2022). Bridging thrombolysis in atrial fibrillation stroke is associated with increased hemorrhagic complications without improved outcomes. J Neurointerv Surg.

[33] Yaghi S, Mistry E, Havenon AD (2021). Effect of alteplase use on outcomes in patients with atrial fibrillation: analysis of the initiation of anticoagulation after cardioembolic stroke study. J Am Heart Assoc.

[34] Chalos V, LeCouffe NE, Uyttenboogaart M (2019). Endovascular treatment with or without prior intravenous alteplase for acute ischemic stroke. J Am Heart Assoc.

[35] Kaesmacher J, Dobrocky T, Heldner MR (2018). Systematic review and meta-analysis on outcome differences among patients with TICI2b versus TICI3 reperfusions: success revisited. J Neurol Neurosurg Psychiatry.

[36] Guo YJ, Yang YQ, Zhou MK (2018). Risk factors of haemorrhagic transformation for acute ischaemic stroke in Chinese patients receiving intravenous recombinant tissue plasminogen activator: a systematic review and meta-analysis. Stroke Vasc Neurol.

[37] Akbik F, Alawieh A, Cawley CM (2021). Differential effect of mechanical thrombectomy and intravenous thrombolysis in atrial fibrillation associated stroke. J Neurointerv Surg.

[38] Zoppo GJD, Poeck K, Pessin MS (1992). Recombinant tissue plasminogen activator in acute thrombotic and embolic stroke. Ann Neurol.

[39] Whitesell RT, Steenburg SD (2014). Imaging findings of acute intravascular thrombus on non-enhanced computed tomography. Emerg Radiol.

[40] Zhou Y, Zhang L, Ospel J (2022). Association of intravenous alteplase, early reperfusion, and clinical outcome in patients with large vessel occlusion stroke: post hoc analysis of the randomized DIRECT-MT trial. Stroke.

[41] Lansberg MG, O’Donnell MJ, Khatri P (2012). Antithrombotic and thrombolytic therapy for ischemic stroke: Antithrombotic Therapy and Prevention of Thrombosis, 9th ed: American College of Chest Physicians Evidence-Based Clinical Practice Guidelines. Chest.

[42] Lees KR, Bluhmki E, von Kummer R (2010). Time to treatment with intravenous alteplase and outcome in stroke: an updated pooled analysis of ECASS, ATLANTIS, NINDS, and EPITHET trials. Lancet.

[43] Derex L, Nighoghossian N (2008). Intracerebral haemorrhage after thrombolysis for acute ischemic stroke: an update. J Neurol Neurosurg Psychiatry.

[44] Chen J, Zeng ZW, Fang ZW (2023). Risk factors for thrombolysis-related intracranial hemorrhage: a systematic review and meta-analysis. Thromb J.

